# Complications and mortality following percutaneous and laparoscopic liver biopsy: A multicenter study in a resource‑limited healthcare system

**DOI:** 10.1371/journal.pone.0347300

**Published:** 2026-04-17

**Authors:** Nadieh Abdallah, Ahed Almahdi, Diana Shella, Rasha Al-Masri, Iyad Maqboul, Mohammad Jaber, Ramzi Shawahna

**Affiliations:** 1 Department of Medicine, Faculty of Medicine and Allied Health Sciences, An-Najah National University, Nablus, Palestine; 2 An-Najah National University Hospital, Nablus, Palestine; 3 Department of Physiology, Pharmacology, and Toxicology, Faculty of Medicine and Allied Health Sciences, An-Najah National University, Nablus, Palestine; 4 Clinical Research Center, An-Najah National University Hospital, Nablus, Palestine; University of Diyala College of Medicine, IRAQ

## Abstract

This study was conducted to assess the incidence and types of complications and mortality following liver biopsy, and to identify independently associated factors that can inform clinical practice in a resource‑limited healthcare system. A retrospective multicenter study was conducted across six major hospitals between January 2020 and December 2025. Medical records of 218 patients undergoing percutaneous and laparoscopic liver biopsies were reviewed. Demographic, clinical, laboratory, procedural, and outcome variables were extracted using a validated data collection form. Inferential analyses were conducted using chi‑square, Fisher’s exact, and Mann‑Whitney U tests, while multivariate logistic regression models were employed to identify factors independently associated with complications and mortality. The most common types of complications were infection (n = 7, 3.2%) and hemorrhage (n = 6, 2.8%), followed by pulmonary complications (n = 4, 1.8%), metabolic disturbances (n = 3, 1.4%), and acute kidney injury (n = 2, 0.9%). Mortality was recorded in 6 patients (2.8%). Higher pre-operative white blood cell count was independently associated with infections (OR: 1.28, 95% CI: 1.02–1.62, p = 0.036). Older age was independently associated with mortality (OR: 1.07 per year increase, 95% CI: 1.01–1.15, p = 0.035). Hemorrhage and pulmonary complications were more frequent after laparoscopic biopsy and under general anesthesia, although these associations did not remain significant in adjusted models. This study provides the first systematic evidence on liver biopsy safety in Palestine, a resource‑limited healthcare system, thereby filling a critical gap in the regional literature. The study identified pre-operative increases in white blood cell count as a predictor of infection and older age as a predictor of mortality. These simple, pragmatic markers can guide monitoring and risk stratification in constrained environments, offering actionable insights for clinicians and policymakers. Future studies should be conducted to evaluate whether these markers can help reduce complications and mortality.

## Introduction

Liver biopsy continues to play an important role in modern clinical practice [1,2]. Liver biopsy remains a cornerstone in hepatology, providing direct histopathological evidence that guides diagnosis, staging, and therapeutic decisions across a wide spectrum of liver diseases [3,4]. Despite advances in non‑invasive imaging and serological markers, biopsy continues to be indicated when clinical uncertainty persists, particularly in cases of unexplained liver enzyme abnormalities, suspected malignancy, or when precise grading of fibrosis and inflammation is required [5,6]. The procedure involves extraction of a small but representative tissue core, most commonly via percutaneous, transjugular, or laparoscopic approaches, each selected according to patient comorbidities and procedural risk [1,6,7]. Modern practice increasingly employs ultrasound or computed tomography (CT) guidance to enhance safety and diagnostic yield, while laparoscopic biopsy offers direct visualization and hemostasis in complex cases [1,6,7]. Thus, liver biopsy continues to occupy a critical role in clinical hepatology, balancing its invasive nature against its unparalleled diagnostic accuracy.

Liver disease represents a major global health challenge, accounting for nearly two million deaths annually and approximately 4% of all global mortality [8,9]. The burden is driven largely by cirrhosis, hepatocellular carcinoma, and complications of viral hepatitis, alcohol‑related liver disease, and metabolic dysfunction‑associated steatotic liver disease [1,6,7,10]. In many low‑ and middle‑income countries (LMICs), including those in the Middle East, the prevalence of chronic liver disease is rising, yet access to advanced non‑invasive diagnostic modalities remains limited [11–13]. In such resource‑constrained settings, liver biopsy retains particular importance as it provides definitive histological information that guides clinical management when imaging and laboratory markers are insufficient [5,14,15]. By directly characterizing fibrosis, inflammation, and neoplastic changes, biopsy offers a level of diagnostic certainty that is indispensable for tailoring treatment strategies and improving patient outcomes in healthcare systems where alternative technologies are scarce.

Although liver biopsy is generally considered safe, it is not without risk. Reported complication rates vary between 0.5% and 5%, with infection and hemorrhage being the most frequent adverse events [7,16]. Minor complications such as transient pain or hematoma are relatively common, while major complications including bile leak, pneumothorax, and injury to adjacent organs are rare but clinically significant [17]. Laboratory changes often accompany these events, with post‑procedure elevations in white blood cell count reflecting inflammatory or infectious processes, and declines in hemoglobin or platelet count signaling bleeding risk. Mortality following liver biopsy is uncommon, estimated at less than 0.1% in large series, but when it occurs, it is typically related to uncontrolled hemorrhage, sepsis, or severe comorbid liver disease [7,16]. These risks underscore the importance of careful patient selection, procedural planning, and post‑biopsy monitoring, particularly in healthcare systems where access to advanced supportive care may be limited.

Persistent gaps in the literature are most pronounced in resource-limited healthcare systems, where evidence on the safety profile of liver biopsy, particularly the spectrum of complications (e.g., hemorrhage, infection, bile leak), procedure-related and post-procedure mortality, and modifiable predictors, remains sparse and inconsistently reported [4,7,16]. While meta-analytic and national database studies have quantified complication incidence and highlighted bleeding risk factors spanning patient-, operator-, and technique-level determinants, these findings largely derive from well-resourced settings and may not generalize to contexts with constrained perioperative support, limited imaging guidance, and variable post-biopsy monitoring capacity [18,19]. In Palestine, where hospitals navigate logistical and infrastructural constraints, defining the types and timing of complications, estimating procedure-related mortality, and identifying pragmatic factors can be essential to inform local guidelines, triage pathways, and risk mitigation strategies. By addressing these knowledge gaps in a resource-limited system, this study aims to deliver actionable evidence that might be used to improve patient selection, procedural planning, and post-biopsy surveillance, ultimately enhancing safety and outcomes [7,18,19].

The objective of this study was to evaluate outcomes and clinical characteristics of patients undergoing liver biopsy (percutaneous and laparoscopic) in Palestinian hospitals, with particular attention to the incidence and types of procedure‑related complications, laboratory changes associated with adverse events, and mortality. We further aimed to identify factors associated with common complications and mortality. By integrating demographic, clinical, laboratory, and procedural variables, this study sought to provide a comprehensive assessment of the safety profile of percutaneous and laparoscopic liver biopsies in a real‑world setting. Importantly, this investigation is the first to systematically analyze liver biopsy outcomes in Palestine, a resource‑limited healthcare system, thereby contributing region‑specific evidence to the broader literature from LMICs. The novelty of this work lies in its dual focus on both common complications and mortality, coupled with the identification of independently associated factors, which can be highly relevant to clinicians working in constrained environments. This unique perspective ensures that the findings are not only scientifically rigorous but also directly applicable to improving patient care in similar healthcare systems worldwide.

## Methods

### Study design and settings

This study was designed as a retrospective observational study conducted in accordance with the Strengthening the Reporting of Observational Studies in Epidemiology (STROBE) checklist (S1 Table). The study reviewed medical records of patients who underwent liver biopsy between January 2020 and December 2025 across major Palestinian hospitals, including An‑Najah National University Hospital (Nablus), Rafidia Hospital (Nablus), Ibn Sina Hospital (Jenin), Palestine Medical Complex (Ramallah), Al‑Makassed Hospital (Jerusalem), and Al‑Ahli Hospital (Hebron). These institutions represent the principal referral centers in the region with the capacity to perform liver biopsy procedures. The Palestinian healthcare system is resource‑limited, with constrained access to advanced diagnostic modalities and perioperative support. Within this fragile infrastructure, liver biopsy is performed infrequently and often under less-than-optimal conditions. Consequently, the complication and mortality rates reported reflect the realities of clinical practice in this setting rather than methodological bias. This context provides a unique opportunity to evaluate the safety and outcomes of percutaneous and laparoscopic liver biopsies in real‑world circumstances where systemic constraints amplify the impact of adverse events.

Data for this study were accessed between 01/01/2025 and 30/12/2025 with formal permission from the participating hospitals. During data collection, the authors had access to medical records solely for research purposes. All identifying information was removed at the time of extraction, and no information that could identify individual participants was retained or reported in this study.

### Study population

The study population consisted of all patients who underwent liver biopsy between January 2020 and December 2025 in the participating hospitals. Patients of all ages and both sexes were eligible, reflecting the diversity of individuals presenting for diagnostic evaluation of liver disease in routine clinical practice. To ensure methodological rigor, only cases with complete perioperative records, including demographic, clinical, laboratory, and procedural data, were included. Patients were excluded if their records lacked essential perioperative information, such as laboratory reports, procedural details, or documentation of survival/mortality status, if they underwent liver transplantation or other major hepatic surgery at the time of biopsy, or if postoperative monitoring and follow‑up documentation were insufficient. This approach allowed the study to capture a comprehensive cohort representative of real‑world practice in a resource‑limited healthcare system, while maintaining data quality standards necessary for reliable analysis.

### Sample size

The study size was determined pragmatically to balance feasibility with the need for sufficient statistical power to evaluate rare outcomes such as complications and mortality. Based on prior reports, we initially aimed to capture approximately 300 consecutive liver biopsy cases across the participating hospitals. During the study period, 290 biopsies were performed (69 laparoscopic and 221 percutaneous). After applying strict inclusion and exclusion criteria to ensure complete perioperative documentation, the final analytic cohort comprised 218 patients.

In routine practice across the participating centers, percutaneous biopsy was more frequently performed because it requires fewer resources, can be conducted under local anesthesia, and is more accessible in resource‑limited hospitals. Laparoscopic biopsy was reserved for patients with complex presentations or when direct visualization was deemed necessary. This distribution reflects the pragmatic nature of the study design and enhances the generalizability of the findings to similar healthcare systems where percutaneous biopsy remains the predominant approach.

Although formal power calculations were constrained by the retrospective design and the rarity of events, the study sought to maximize statistical power by including all consecutive cases across six referral centers. To minimize the risk of overfitting, the number of predictors in the regression models was restricted to variables with strong clinical plausibility and sufficient event counts. To minimize the risk of overfitting, the number of predictors in the regression models was restricted to variables with strong clinical plausibility and sufficient event counts. Diagnostics were used to evaluate performance of the regression models.

### Variables and data collection

Data were extracted retrospectively from hospital records using a standardized collection form to ensure consistency across centers. Variables encompassed demographic, clinical, laboratory, procedural, and outcome domains. Demographic and clinical history variables included patient age (recorded in years as a continuous variable), sex (male or female), smoking status (yes/no), and presence of comorbidities (yes/no). Prior diagnosis of liver disease was noted when documented in medical records. Procedural variables captured the type of biopsy performed (laparoscopic or percutaneous), guidance modality (direct vision, ultrasound, or computed tomography), and anesthesia type (general or local). Pre‑biopsy imaging was recorded as a binary variable (yes/no). Indications for biopsy were categorized according to clinical documentation, including abnormal imaging findings, suspicion of malignancy, abnormal liver function tests, hepatomegaly, jaundice, screening, and other less frequent indications. Laboratory parameters were collected both before and after the procedure. These included white blood cell count (×10^3^/µL), platelet count (×10^3^/µL), hemoglobin (g/dL), aspartate aminotransferase (U/L), alanine aminotransferase (U/L), alkaline phosphatase (U/L), gamma‑glutamyl transferase (U/L), total bilirubin (mg/dL), prothrombin time (seconds), and international normalized ratio (INR). Clinical impression prior to biopsy was extracted from physician notes and categorized into malignancy, hepatitis, cirrhosis, fatty liver, cholestatic liver disease, metabolic liver disease, fibrosis, or other suspected conditions. Final histopathological diagnosis was recorded from pathology reports and classified into malignancy, hepatitis, fatty liver, cirrhosis, cholestatic liver disease, fibrosis, metabolic liver disease, or normal findings. Complications and perioperative outcomes were documented as binary variables (yes/no) and further categorized by type (infection, hemorrhage, pulmonary, metabolic, acute kidney injury, or other). Timing of complications was noted as immediate or delayed. Observation patterns included discharge within hours or ward transfer. Post‑biopsy imaging was recorded when performed. Mortality was defined as death occurring during the perioperative period or subsequent follow‑up. Survival status was determined using the electronic health system, which records death certificates for deceased patients and recent follow‑up documentation for those alive at the time of data collection. For the purposes of this study, mortality was reported as all‑cause mortality during the perioperative or follow‑up period, without adjudication of whether death was directly procedure‑related or due to progression of underlying disease.

To ensure methodological rigor, the data collection form was subjected to expert review prior to use. Surgeons, hepatologists, pathologists, and internists independently examined the form to confirm that all relevant clinical, laboratory, and procedural variables were appropriately captured. In addition, the research team, comprising surgeons, anesthesiologists, and final medical students, evaluated the form for clarity, feasibility, and completeness in the context of retrospective chart review. Through this iterative process, both face validity (appropriateness and relevance of items) and content validity (comprehensiveness of coverage) were established, thereby strengthening the reliability of the extracted data. The final data collection form is provided in S2 Table.

### Data analysis

Data entry, coding, and statistical analysis were performed using IBM SPSS (version 21.0; IBM, Armonk, NY, USA) and R software (version 3.2.5; R Foundation for Statistical Computing, Vienna, Austria). Descriptive statistics were used to summarize demographic, clinical, laboratory, and procedural variables. Continuous variables were expressed as medians with interquartile range [Q1, Q3], while categorical variables were reported as frequencies (n) and percentages (%). For inferential analysis, comparisons between patients with and without complications or mortality were performed using the Mann-Whitney U test for continuous variables, given their non‑normal distribution, and chi‑square or Fisher’s exact tests for categorical variables. Statistical significance was defined as a two‑sided p‑value < 0.05.

Multivariate logistic regression models were built to identify factors independently associated with complications and mortality. Because both complications and mortality were relatively rare events, logistic regression models were built retaining clinically plausible predictors to minimize small‑sample bias. Caution was applied to ensure producing narrow 95% confidence intervals (CIs) and stable estimates. Model diagnostics were used to assess performance. Discrimination was evaluated using the area under the receiver operating characteristic curve (AUC) and calibration was assessed with the Hosmer-Lemeshow goodness‑of‑fit test. Together, these diagnostics assessed statistical robustness and clinical interpretability of the identified factors.

### Ethical considerations

This study was conducted in accordance with the ethical principles outlined in the Declaration of Helsinki. Approval was obtained from the Institutional Review Board (IRB) of An‑Najah National University (approval #: Med. Dec.2024/61), as well as authorization from the Palestinian Ministry of Health to access patient records across participating hospitals. Given the retrospective design and reliance on existing medical records, the IRB of An‑Najah National University waived the requirement for written informed consent. Confidentiality and privacy were strictly maintained throughout the study; all patient identifiers were removed during data extraction, and access to raw data was restricted to the research team.

## Results

### Demographic, clinical history, and procedural variables

The case selection process is illustrated in S1 Fig. During the study period, a total of 290 liver biopsies were identified across the participating hospitals (69 laparoscopic and 221 percutaneous). After excluding 72 cases with incomplete documentation, 218 patients remained eligible and were included in the final analysis. The cohort comprised118 males (54.1%) and 100 females (45.9%), with a median age of 52.0 years [35.0, 63.0] (Table 1). Smoking was reported in 86 patients (39.4%), and comorbidities were present in 170 (78%). Fifty patients (22.9%) had a previously established diagnosis of liver disease, and pre-biopsy imaging was performed in 190 cases (87.2%). Regarding procedural characteristics, 64 patients (29.4%) underwent laparoscopic biopsy and 154 (70.6%) percutaneous biopsies. Guidance modalities included direct vision in 62 cases (28.4%), computed tomography in 63 (28.9%), and ultrasound in 93 (42.7%). General anesthesia was administered in 63 patients (28.9%), whereas 155 (71.1%) received local anesthesia. Detailed baseline characteristics are presented in Table 1.

**Table 1:** Baseline demographic clinical history, and procedural variables of patients undergoing liver biopsy (*n = 218*).

Variable	n (%) or median [Q1, Q3]
Demographics	
Sex	
Male, n (%)	118 (54.1)
Female, n (%)	100 (45.9)
Age (years), median [Q1, Q3]	52.0 [35.0, 63.0]
Smoking status, (yes), n (%)	86 (39.4)
Clinical history	
Comorbidities, (yes), n (%)	170 (78)
Prior liver disease, (yes), n (%)	50 (22.9)
Procedural variables	
Imaging before biopsy, (yes)	190 (87.2)
Type of biopsy procedure	
Laparoscopic, n (%)	64 (29.4)
Percutaneous, n (%)	154 (70.6)
Type of guidance	
Direct vision, n (%)	62 (28.4)
Computed tomography, n (%)	63 (28.9)
Ultrasound, n (%)	93 (42.7)
Type of anesthesia	
General, n (%)	63 (28.9)
Local, n (%)	155 (71.1)

Q1: lower quartile, Q3: upper quartile.

### Indications for biopsy

The most common indication for biopsy was abnormal imaging findings, observed in 117 patients (53.7%) (S3 Table). Suspicion of malignancy accounted for 57 cases (26.1%), while abnormal liver function tests prompted biopsy in 54 patients (24.8%). Other indications included unexplained hepatomegaly (18, 8.3%), unspecified jaundice (n = 11, 5.0%), and screening (n = 8, 3.7%). Less frequent motives included hepatosplenomegaly (n = 5, 2.3%), non-specific liver disease (n = 3, 1.4%), and rare scenarios such as failed trans-jugular or CT-guided biopsy (each 1, 0.5%).

### Pre-procedural laboratory evaluation

Baseline laboratory evaluation revealed a median white blood cell count of 6.7 × 10^3^/µL [4.9, 8.7], platelet count of 226.0 × 10^3^/µL [138.5, 294.0], and hemoglobin of 12.4 g/dL [10.8, 14.0] (S4 Table). Liver function tests demonstrated elevated median aspartate aminotransferase of 40.3 U/L [23.6, 72.8], alanine aminotransferase of 33.5 U/L [15.9, 67.1], alkaline phosphatase of 140.0 U/L [86.4, 260.0], and gamma-glutamyl transferase of 119.0 U/L [61.5, 175.0]. The median total bilirubin was 0.7 mg/dL [0.4, 2.6], with a median prothrombin time of 14.0 seconds [13.0, 15.6] and international normalized ratio of 1.1 [1.0, 1.2].

### Clinical impression prior to biopsy and final histopathological diagnosis

The initial clinical impression most frequently suggested malignancy (n = 115, 52.8%), followed by hepatitis (n = 35, 16.1%) and cirrhosis (n = 34, 15.6%) (S5 Table). Final histopathological results confirmed malignancy in 95 patients (43.6%), hepatitis in 40 (18.3%), fatty liver in 29 (13.3%), cirrhosis in 26 (11.9%), cholestatic liver disease in 9 (4.1%), fibrosis in 7 (3.2%), metabolic liver disease in 1 (0.5%), and normal findings in 11 (5.0%).

The final biopsy results demonstrated that malignancy was the most frequent diagnosis, identified in 95 patients (43.6%) (S2 Fig). Hepatitis was confirmed in 40 cases (18.3%), fatty liver in 29 (13.3%), and cirrhosis in 26 (11.9%). Less common findings included cholestatic liver disease in 9 patients (4.1%), fibrosis in 7 (3.2%), and metabolic liver disease in 1 (0.5%). Normal histology was observed in 11 patients (5.0%).

### Post-procedure laboratory values, complications, and perioperative outcomes

Post-procedure laboratory values demonstrated a median white blood cell count of 7.9 × 10^3^/µL [5.7, 11.1], platelet count of 224.0 × 10^3^/µL [141.0, 295.0], and hemoglobin of 11.5 g/dL [9.8, 13.5] (S6 Table). Liver enzymes remained elevated, with a median alanine aminotransferase of 36.5 U/L [16.8, 70.0], alkaline phosphatase of 129.0 U/L [87.3, 280.5], and gamma-glutamyl transferase of 96.0 U/L [40.8, 213.5]. The median total bilirubin was 0.8 mg/dL [0.4, 2.5].

Procedure-related complications occurred in 17 patients (7.8%) (Table 2). The most frequent were infection (n = 7, 3.2%) and hemorrhage (n = 6, 2.8%), followed by pulmonary complications (n = 4, 1.8%), metabolic disturbances (n = 3, 1.4%), and acute kidney injury (n = 2, 0.9%). Other complications were reported in 2 patients (0.9%). Immediate complications occurred in 6 cases (35.3%), while delayed complications were observed in 11 (64.7%). Most patients (n = 163, 74.8%) were discharged within hours post-procedure, whereas 55 (25.2%) required ward transfer. Post-biopsy imaging was performed in 12 patients (5.5%). Mortality was recorded in 6 cases (2.8%). Detailed perioperative outcomes are shown in Table 2.

**Table 2:** Complications and perioperative outcomes following liver biopsy.

Variable	n (%)
Procedure associated complications, (yes)	17 (7.8)
Complication type	
Infection	7 (3.2)
Hemorrhage	6 (2.8)
Pulmonary	4 (1.8)
Metabolic	3 (1.4)
Acute kidney injury	2 (0.9)
Other	2 (0.9)
Timing of complication	
Immediate	6 (35.3)
Delayed	11 (64.7)
Duration of observation	
Hours post-procedure before discharge	163 (74.8)
Ward transfer	55 (25.2)
Post-biopsy imaging performed, (yes)	12 (5.5)
Mortality, (yes)	6 (2.8)

### Factors associated with outcomes

#### Factors associated with infections.

Bivariate analysis showed that patients who suffered infections demonstrated distinct perioperative features compared to those without infection (S7 Table). Pre‑procedure white blood cell counts were significantly higher in infected patients (median 9.5 × 10^3^/µL [8.1, 12.0] vs. 6.5 × 10^3^/µL [4.9, 8.5], p-value = 0.036). Similarly, liver enzymes including aspartate aminotransferase and alanine aminotransferase were also elevated (195.0 U/L [101.0, 209.0] vs. 38.4 U/L [23.4, 60.4], p = 0.008 for aspartate aminotransferase and 165.0 U/L [58.0, 345.0] vs. 32.7 U/L [15.7, 60.0], p-value = 0.021 for alanine aminotransferase). All infections occurred following percutaneous biopsy, whereas no cases were observed after laparoscopic biopsy. Other demographic, clinical, and laboratory variables did not show statistically significant associations with infection.

In the multivariate logistic regression model (Table 3), perioperative white blood cell count remained independently associated with infection. Each unit increase in white blood cell count was linked to higher odds of infection (B = 0.25, SE = 0.12, Wald = 4.38, p = 0.036; OR = 1.28, 95% CI: 1.02–1.62). Aspartate aminotransferase and alanine aminotransferase did not reach statistical significance in the adjusted model. The diagnostic measures indicated that this model had acceptable performance. The Nagelkerke R^2^ was 0.252, suggesting the model explained about a quarter of the variance in infection occurrence, which is reasonable for clinical observational data. The Hosmer-Lemeshow goodness‑of‑fit test was non‑significant (χ^2^ = 3.449, df = 7, p = 0.841), indicating good calibration between predicted and observed outcomes. Overall, the model demonstrated adequate fit and discrimination, supporting its reliability for identifying factors independently associated with infection in this cohort.

**Table 3:** Factors independently associated with complication categories and mortality.

						95% CI for OR	
Factor	B	SE	Wald	p	OR	Lower	Upper
**Infections**							
White blood cells (×10^3^/µL)	0.25	0.12	4.38	**0.036**	1.28	1.02	1.62
Aspartate aminotransferase (U/L)	−0.01	0.01	1.08	0.300	0.99	0.98	1.01
Alanine aminotransferase (U/L)	0.01	0.01	2.45	0.118	1.01	1.00	1.02
Constant	−5.40	1.46	13.73	**< 0.001**	0.00		
**Hemorrhage**							
Type of biopsy procedure (laparoscopic vs. percutaneous)	1.49	0.89	2.81	0.094	4.44	0.78	25.41
Platelet count (×10^3^/µL)	0.00	0.00	0.00	0.972	1.00	0.99	1.01
Hemoglobin (g/dL)	−0.15	0.20	0.57	0.449	0.86	0.58	1.28
Constant	−2.40	2.62	0.83	0.361	0.09		
**Pulmonary complications**							
Type of biopsy procedure (laparoscopic vs. percutaneous)	0.86	1.26	0.46	0.496	2.36	0.20	27.81
Hemoglobin (g/dL)	−0.49	0.29	2.74	0.098	0.62	0.35	1.09
Aspartate aminotransferase (U/L)	−0.02	0.04	0.17	0.679	0.98	0.92	1.06
Alanine aminotransferase (U/L)	0.00	0.03	0.00	0.991	1.00	0.94	1.06
Constant	2.52	3.36	0.56	0.454	12.38		
**Mortality**							
Age (years)	0.11	0.05	4.77	**0.029**	1.12	1.01	1.23
Type of biopsy procedure (laparoscopic vs. percutaneous)	−1.58	1.26	1.57	0.210	0.21	0.02	2.45
Alanine aminotransferase (U/L)	0.00	0.00	0.18	0.671	1.00	1.00	1.01
Constant	−8.72	3.35	6.76	**0.009**	0.00		

CI: confidence interval, OR: odds ratio, SE: standard error, p: p-value, statistically significant p-values are in boldface.

#### Factors associated with hemorrhage.

Bivariate analysis showed that patients who suffered hemorrhage demonstrated distinct procedural features compared to those without hemorrhage (S8 Table). Hemorrhage was significantly more frequent following laparoscopic biopsy (4 of 64, 6.3%) compared to percutaneous biopsy (2 of 154, 1.3%; p = 0.042). Similarly, patients who underwent general anesthesia experienced higher rates of hemorrhage (4 of 63, 6.3%) than those receiving local anesthesia (2 of 155, 1.3%; p = 0.038). Other demographic, clinical, and laboratory variables did not show statistically significant associations with hemorrhage.

In the multivariate logistic regression model (Table 3), the type of biopsy procedure (laparoscopic vs. percutaneous) showed a non‑significant association with hemorrhage (B = 1.49, SE = 0.89, Wald = 2.81, p = 0.094; OR = 4.44, 95% CI: 0.78–25.41). Platelet count and hemoglobin levels were not independently associated with hemorrhage (p = 0.972 and p = 0.449, respectively). These findings suggest that while laparoscopic biopsy demonstrated a trend toward higher odds of bleeding, no variable remained statistically associated with hemorrhage after adjustment. The diagnostic measures indicated that this model was limited but acceptable. The Nagelkerke R^2^ was 0.078, indicating that the model explained only a small proportion of the variance in hemorrhage occurrence, which is expected given the very low number of events. The Hosmer-Lemeshow test was non‑significant (χ^2^ = 5.268, df = 8, p = 0.729), showing good calibration between observed and predicted outcomes. Overall, while the model fit the data adequately, its explanatory power was modest, reflecting the rarity of hemorrhage events in the cohort.

#### Factors associated with pulmonary complications.

Bivariate analysis showed that patients who developed pulmonary complications demonstrated distinct procedural features compared to those without pulmonary events (S9 Table). Pulmonary complications were significantly more frequent following laparoscopic biopsy (3 of 64, 4.7%) compared to percutaneous biopsy (1 of 154, 0.6%; p = 0.043). Similarly, patients who underwent general anesthesia experienced higher rates of pulmonary complications (3 of 63, 4.8%) than those receiving local anesthesia (1 of 155, 0.6%; p = 0.040). Other demographic, clinical, and laboratory variables did not show statistically significant associations, although lower hemoglobin and reduced aspartate aminotransferase levels demonstrated borderline trends.

In the adjusted logistic regression model (Table 3), the type of biopsy procedure (laparoscopic vs. percutaneous) showed a non‑significant association with pulmonary complications (B = 1.49, SE = 0.89, Wald = 2.81, p = 0.094; OR = 4.44, 95% CI: 0.78–25.41). Platelet count and hemoglobin levels were also not independently associated (p = 0.972 and p = 0.449, respectively). These findings suggest that although laparoscopic biopsy showed a trend toward higher odds of pulmonary complications, no variable remained statistically associated after adjustment. The diagnostic measures indicated that the model fit was acceptable. The Nagelkerke R^2^ was 0.205, indicating that the model explained about 20% of the variance in pulmonary events, which is reasonable given the small number of cases. The Hosmer-Lemeshow test was non‑significant (χ^2^ = 3.163, df = 7, p = 0.870), showing good calibration between predicted and observed outcomes. Overall, the model demonstrated adequate fit and reliability, though its explanatory power was modest, reflecting the limited number of pulmonary complications in the cohort.

#### Factors associated with mortality.

Bivariate analysis showed that patients who died following biopsy demonstrated distinct clinical and laboratory features compared to survivors (S10 Table). Mortality was significantly associated with older age (median 65.5 years [56.0–69.0] vs. 52.0 years [35.0–62.0], p = 0.035). Several procedure‑related complications were strongly linked to death, including infection (3 of 6 deaths, 50.0% vs. 4 of 212 survivors, 1.8%; p < 0.001), pulmonary complications (2 of 6 deaths, 33.3% vs. 2 of 212 survivors, 0.9%; p < 0.001), metabolic disturbances (2 of 6 deaths, 33.3% vs. 1 of 212 survivors, 0.5%; p < 0.001), and acute kidney injury (2 of 6 deaths, 33.3% vs. 0 of 212 survivors, 0.0%; p < 0.001). Mortality was also significantly associated with post‑biopsy imaging (3 of 6 deaths, 50.0% vs. 9 of 212 survivors, 4.1%; p < 0.001). In terms of laboratory predictors, non‑survivors had markedly higher pre‑procedure alkaline phosphatase (median 452.0 U/L [321.0, 680.0] vs. 133.0 U/L [86.2, 210.0], p = 0.008). Similarly, non‑survivors had markedly higher post‑procedure white blood cell counts (median 12.6 × 10^3^/µL [11.5–15.0] vs. 7.7 × 10^3^/µL [5.6–10.8], p = 0.004), elevated alanine aminotransferase (79.5 U/L [74.2–104.0] vs. 34.7 U/L [16.0–59.5], p = 0.033), alkaline phosphatase (452.0 U/L [427.0–552.0] vs. 121.0 U/L [86.3–237.0], p = 0.004), and gamma‑glutamyl transferase (445.0 U/L [410.5–513.5] vs. 86.3 U/L [40.6–144.0], p = 0.019). Other demographic, clinical, and laboratory variables did not show statistically significant associations with mortality.

In the adjusted logistic regression model (Table 3), age remained independently associated with mortality. Each additional year of age was linked to higher odds of death (B = 0.11, SE = 0.05, Wald = 4.77, p = 0.029; OR = 1.12, 95% CI: 1.01–1.23). The type of biopsy procedure (laparoscopic vs. percutaneous) showed no significant association (p = 0.210; OR = 0.21, 95% CI: 0.02–2.45). Alanine aminotransferase levels were also not independently associated (p = 0.671). These findings indicate that older age was consistently associated with mortality, while biopsy type and liver enzyme levels did not show independent associations after adjustment. The diagnostic measures indicated that this model had acceptable fit and moderate explanatory power. The Nagelkerke R^2^ was 0.318, suggesting that the model explained about one‑third of the variance in mortality, which is relatively strong compared to the other complication models. The Hosmer-Lemeshow test was non‑significant (χ^2^ = 8.724, df = 8, p = 0.366), confirming good calibration between predicted and observed outcomes. Overall, the model demonstrated reliable fit and reasonable discrimination, supporting its validity for identifying factors independently associated with mortality in this cohort.

## Discussion

Complications and mortality following liver biopsy remain critical concerns in hepatology, particularly in settings where supportive care resources are limited [5,7,18,19]. While systematic assessments of liver biopsy safety have been reported from other LMICs [20–22], to our knowledge, this is the first multicenter study from Palestine to systematically evaluate the safety profile of laparoscopic and percutaneous liver biopsy within this fragile and resource‑limited healthcare system. Stratified analyses revealed that infectious complications were confined to percutaneous procedures, whereas hemorrhagic and pulmonary events were more frequently associated with laparoscopic biopsy. Mortality occurred in both groups but was predominantly linked to percutaneous procedures. Beyond these procedural differences, regression analyses showed that higher pre-operative white blood cell count was independently associated with infectious complications, while older age was independently associated with mortality. Together, bivariate and multivariate regression highlight that both procedural approach and perioperative clinical parameters shape the risk profile of liver biopsy in resource‑constrained settings. These findings are significant because they provide locally relevant evidence that might be used to guide clinicians in patient selection, perioperative monitoring, and risk stratification. They are also particularly informative for hepatologists, surgeons, anesthesiologists, and policymakers working in resource‑constrained environments, where biopsy remains indispensable yet carries heightened risks.

Large multicenter studies and meta‑analyses conducted in well‑resourced countries consistently reported the rates of major complications and mortality following liver biopsy [5,7,18,19]. For instance, a nationwide German database analysis of image‑guided biopsies found major complications in only 1.6% (195 out of 12,117 biopsies) of cases and mortality in 0.02% (3 deaths out of 12,117 biopsies) [7], while an Italian multicenter study reported complication rates of 1.4% of 1,838 patients (26 cases) and no procedure‑related deaths [23]. Evidence from LMICs also illustrates variability in outcomes [20–22]. An audit study from Pakistan reported a 5% overall complication rate, including 1.1% major complications and no mortality, while a study from Egypt documented a 3.6% overall complication rate with no procedure‑related deaths [20,21]. In contrast, our cohort demonstrated higher complication and mortality rates than those reported in large series from other countries. These differences might also be understood as reflections of system‑level constraints rather than intrinsic procedural risk. It is important to note that Palestinian hospitals operate under fragile infrastructure, where imaging guidance may be inconsistent, perioperative monitoring less intensive, and access to advanced supportive care restricted. Such limitations can magnify the clinical impact of adverse events that might otherwise be manageable. Although comprehensive chart reviews and standardized definitions were applied across centers to minimize reporting bias, the elevated complication rates observed in our cohort might reflect a combination of factors, including differences in case‑mix, procedural practices, and the limited perioperative infrastructure available in Palestinian hospitals. Therefore, these rates should not be interpreted as evidence that liver biopsy is inherently unsafe.

In this study, we did not only report aggregate complication and mortality rates, rather, we stratified outcomes by complication category and biopsy procedure and examined independently associated factors for each complication category as well as for mortality. The predictors identified in this study are biologically plausible and clinically meaningful. Mortality was independently associated with older age, consistent with prior evidence that advanced age increases vulnerability to perioperative complications and worsens outcomes after invasive procedures [24]. This finding underscores the importance of careful patient selection and heightened surveillance in elderly populations undergoing liver biopsy procedures. Infections were independently predicted by pre-operative increases in white blood cell count, aligning with reports that post‑biopsy leukocytosis can signal sepsis or abscess formation [22,25]. Taken together, these factors emphasize that both simple clinical observations and routine laboratory markers might provide valuable signals of risk. Their relevance is particularly pronounced in resource‑limited settings, where complex scoring systems may be impractical and where actionable, low‑cost indicators are essential for guiding surveillance and intervention. Notably, while laparoscopic biopsy was more often associated with hemorrhagic and pulmonary complications and percutaneous biopsy with infectious complications and mortality, overall complication and mortality rates did not differ significantly between approaches. This suggests that system‑level constraints, rather than biopsy type alone, drive the elevated rates observed in our cohort. These insights might provide a foundation for pragmatic recommendations, including strengthening perioperative monitoring, prioritizing elderly patients and those with abnormal laboratory shifts for closer surveillance, and expanding access to ultrasound‑guided biopsy might reduce complications and improve patient outcomes events [3,4,14,15,17]. At the policy level, investment in training, imaging equipment, and post‑procedure monitoring capacity might be essential to mitigate risks and improve outcomes in resource‑limited healthcare systems. It is important to note that future studies are still needed to evaluate the effects of applying these measures on the incidence of complications and mortality in the Palestinian and other resource-limited healthcare systems. Taken together, our results highlight context‑specific vulnerabilities and provide actionable evidence to guide clinicians in resource‑limited settings, while also pointing to areas where investment in infrastructure and perioperative protocols could substantially improve safety standards.

### Strengths and limitations of the study

This study has a number of notable strengths. First, it represents the first multicenter investigation in Palestine, providing novel evidence from a resource‑limited healthcare system where data on liver biopsy safety have been scarce. Second, the inclusion of six major referral hospitals enhances the generalizability of the findings across diverse institutional settings. Third, the study employed a rigorously validated data collection form, reviewed by surgeons, pathologists, internists, anesthesiologists, and medical students, thereby ensuring both face and content validity. Fourth, the comprehensive capture of demographic, clinical, laboratory, procedural, and outcome variables allowed for a multidimensional analysis of risk factors. Fifth, the regression models addressed the statistical challenges posed by rare events. By restricting factors to variables with strong clinical plausibility and sufficient event counts, the models produced acceptable confidence intervals and interpretable estimates. Sixth, the study not only quantified complications and mortality rates but also identified pragmatic factors through regression analysis, including pre-operative increases in white blood cell count as a marker of infection and older age as a predictor of mortality. Together, these strengths underscore the methodological rigor and clinical relevance of the work.

Nonetheless, some limitations should be acknowledged. First, the retrospective design inherently relies on the accuracy and completeness of medical records, which may introduce information bias. Second, the relatively small number of events (complications and deaths) limited statistical power. Therefore, we acknowledge that the limited sample size and low number of outcome events increase the risk of statistical imprecision and potential overfitting in regression models. These constraints are inherent to the Palestinian healthcare system, where liver biopsy procedures are limited and not routinely performed. In this study, standard diagnostic parameters were used to assess the reliability and stability of the models. Although the estimates should be interpreted with caution, they provide critical locally relevant evidence that might inform clinical practice and guide future research. In addition, we also acknowledge that the complications and mortality rates reported here are higher than those typically described in the literature. These elevated figures should be interpreted cautiously, as they reflect the constraints of a resource‑limited healthcare system rather than intrinsic procedural risk. Although, case definitions were standardized across centers; nonetheless, replication in larger cohorts and across diverse settings remains essential to confirm the generalizability of these findings. Moreover, mortality in this study was reported as events occurring during the perioperative period or subsequent follow‑up. Therefore, mortality in this study can be considered as all‑cause mortality. Therefore, some mortality may have reflected progression of advanced underlying disease rather than procedure‑related complications, although deaths were temporally associated with biopsy. This distinction could not be reliably adjudicated in the retrospective design, and therefore caution is warranted when interpreting mortality estimates. Third, the study did not capture operator‑level variables (e.g., experience, professional training), which may influence complication rates. Fourth, follow‑up duration was variable and relatively short, potentially underestimating late complications. Fifth, the findings may not be directly generalizable to well‑resourced healthcare systems, where infrastructure, monitoring, and supportive care differ substantially. Finally, the study period (January 2020-December 2025) overlapped with the COVID‑19 pandemic. Although liver biopsies are rare in the Palestinian healthcare system, we cannot exclude the possibility that the pandemic influenced procedural volumes, patient acuity, hospital capacity, and complication rates. This potential impact should be considered when interpreting our findings. These limitations, while important, do not diminish the value of the study; rather, they highlight areas for future prospective, larger‑scale investigations.

## Conclusion

This multicenter study is the first to systematically evaluate complications and mortality following liver biopsy in Palestine, a resource‑limited healthcare system. Complication and mortality rates were higher than those reported in well‑resourced settings, underscoring the impact of infrastructural constraints on patient safety. Beyond quantifying adverse outcomes, the study identified higher pre-operative white blood cell count as a factor independently associated with infections and older age as a factor independently associated with mortality. These simple, pragmatic factors are biologically plausible, clinically relevant, and readily available in routine practice, making them particularly valuable in constrained environments where complex risk scores are impractical. The findings provide evidence to inform local practice and policy, while also contributing to global understanding of biopsy safety in resource‑limited healthcare systems. Future studies should explore the utility of these markers in guiding perioperative monitoring, refining patient selection, and ultimately reducing the incidence of complications and mortality in resource‑limited healthcare systems.

## Supporting information

S1 Table Adherence to the STROBE Statement. (DOCX)

S2 Table Data collection form. (DOCX)

S3 Table Clinical indications for liver biopsy. (DOCX)

S4 Table Laboratory parameters prior to liver biopsy. (DOCX)

S5 Table Clinical impression prior to biopsy. (DOCX)

S6 Table Laboratory parameters following liver biopsy. (DOCX)

S7 Table Bivariate analysis of factors associated with procedure-related infections. (DOCX)

S8 Table Bivariate analysis of factors associated with procedure-related hemorrhage. (DOCX)

S9 Table Bivariate analysis of factors associated with procedure-related pulmonary complications. (DOCX)

S10 Table Bivariate analysis of factors associated with mortality. (DOCX)

S1 Data Anonymized raw data. (XLSX)

S1 Fig Flow diagram of patient inclusion process. (TIF)

S2 Fig Distribution of final biopsy diagnoses among study patients. (TIF)

## Abbreviations:

AUC: Area under the receiver operating characteristic curve
CI: Confidence interval
CT: Computed tomography
INR: International normalized ratio
IRB: Institutional Review Board
LMICs: Low‑ and middle‑income countries
OR: Odds ratio
p: p-value
Q1: Lower quartile
Q3: Upper quartile
SE: Standard error
STROBE: Strengthening the Reporting of Observational Studies in Epidemiology
