# Supplementary material for: Complications and mortality following percutaneous and laparoscopic liver biopsy: A multicenter study in a resource‑limited healthcare system
Source: PLoS One. 2026 Apr 17;21(4):e0347300. doi: 10.1371/journal.pone.0347300 (PMC13089758; doi:10.1371/journal.pone.0347300)
Supplement: S2 Table — (DOCX) [file pone.0347300.s002.docx]

**S2 Table. Data collection form.**

| **Section** | **Variable** | **Response Options / Field** |
| --- | --- | --- |
| **A. Demographics** | Patient ID (study code) | ________ |
|  | Age (years) | ________ |
|  | Sex | ☐ Male ☐ Female |
|  | Smoking status | ☐ Yes ☐ No |
| **B. Clinical History** | Comorbidities | ☐ Yes ☐ No |
|  | Specify comorbidities | __________________________ |
|  | Prior diagnosis of liver disease | ☐ Yes ☐ No |
|  | Type of liver disease | ☐ Malignancy ☐ Hepatitis ☐ Cirrhosis ☐ Fatty liver ☐ Cholestatic ☐ Metabolic ☐ Fibrosis ☐ Other: ________ |
| **C. Procedural Variables** | Type of biopsy | ☐ Percutaneous ☐ Laparoscopic |
|  | Guidance modality | ☐ Direct vision ☐ Ultrasound ☐ CT |
|  | Type of anesthesia | ☐ General ☐ Local |
|  | Pre‑biopsy imaging performed | ☐ Yes ☐ No |
|  | Indication for biopsy | ☐ Abnormal imaging ☐ Suspicion of malignancy ☐ Abnormal LFTs ☐ Hepatomegaly ☐ Jaundice ☐ Screening ☐ Other: ________ |
|  | Simultaneous surgery | ☐ Yes ☐ No |
| **D. Laboratory Parameters (Pre‑procedure)** | WBC (×10^3^/µL) | ________ |
|  | Platelet count (×10^3^/µL) | ________ |
|  | Hemoglobin (g/dL) | ________ |
|  | AST (U/L) | ________ |
|  | ALT (U/L) | ________ |
|  | Alkaline phosphatase (U/L) | ________ |
|  | GGT (U/L) | ________ |
|  | Total bilirubin (mg/dL) | ________ |
|  | Prothrombin time (sec) | ________ |
|  | INR | ________ |
| **D. Laboratory Parameters (Post‑procedure)** | WBC (×10^3^/µL) | ________ |
|  | Platelet count (×10^3^/µL) | ________ |
|  | Hemoglobin (g/dL) | ________ |
|  | ALT (U/L) | ________ |
|  | Alkaline phosphatase (U/L) | ________ |
|  | GGT (U/L) | ________ |
|  | Total bilirubin (mg/dL) | ________ |
| **Calculated Δ change** | Δ WBC | ________ |
|  | Δ Platelets | ________ |
|  | Δ Hemoglobin | ________ |
|  | Δ ALT | ________ |
|  | Δ Alkaline phosphatase | ________ |
|  | Δ GGT | ________ |
|  | Δ Bilirubin | ________ |
| **E. Clinical Impression & Histopathology** | Clinical impression prior to biopsy | ☐ Malignancy ☐ Hepatitis ☐ Cirrhosis ☐ Fatty liver ☐ Cholestatic ☐ Metabolic ☐ Fibrosis ☐ Other: ________ |
|  | Final histopathological diagnosis | ☐ Malignancy ☐ Hepatitis ☐ Cirrhosis ☐ Fatty liver ☐ Cholestatic ☐ Metabolic ☐ Fibrosis ☐ Normal |
| **F. Outcomes** | Complications | ☐ Yes ☐ No |
|  | Type of complication | ☐ Infection ☐ Hemorrhage ☐ Pulmonary ☐ Metabolic ☐ AKI ☐ Other: ________ |
|  | Timing of complication | ☐ Immediate ☐ Delayed |
|  | Observation pattern | ☐ Discharge within hours ☐ Ward transfer |
|  | Post‑biopsy imaging performed | ☐ Yes ☐ No |
|  | Mortality | ☐ Yes ☐ No |
