# Supplementary material for: Complications and mortality following percutaneous and laparoscopic liver biopsy: A multicenter study in a resource‑limited healthcare system
Source: PLoS One. 2026 Apr 17;21(4):e0347300. doi: 10.1371/journal.pone.0347300 (PMC13089758; doi:10.1371/journal.pone.0347300)
Supplement: S3 Table — (DOCX) [file pone.0347300.s003.docx]

**S3 Table. Clinical indications for liver biopsy.**

| **Indication** | **n (%)** |
| --- | --- |
| Abnormal imaging findings | 117 (53.7) |
| Suspicion of malignancy | 57(26.1) |
| Abnormal liver function tests | 54 (24.8) |
| Unexplained hepatomegaly | 18 (8.3) |
| Unspecified jaundice | 11 (5.0) |
| Screening | 8 (3.7) |
| Simultaneously during other surgery | 6 (2.8) |
| Hepatosplenomegaly | 5 (2.3) |
| Non-specific liver disease | 3 (1.4) |
| Anti-smooth muscle antibodies (ASMA +ve) | 1 (0.5) |
| Positive IgG and IgM results | 1 (0.5) |
| Received liver transplantation | 1 (0.5) |
| Recurrent loss of consciousness | 1 (0.5) |
| Isolated direct hyperbilirubinemia | 1 (0.5) |
| Failed trans-jugular biopsy | 1 (0.5) |
| Failed CT guided liver biopsy | 1 (0.5) |
| Referred from another hospital | 1 (0.5) |
| Re-evaluation | 1 (0.5) |
| Doubt of diagnosis | 1 (0.5) |
