# Supplementary material for: Complications and mortality following percutaneous and laparoscopic liver biopsy: A multicenter study in a resource‑limited healthcare system
Source: PLoS One. 2026 Apr 17;21(4):e0347300. doi: 10.1371/journal.pone.0347300 (PMC13089758; doi:10.1371/journal.pone.0347300)
Supplement: S4 Table — (DOCX) [file pone.0347300.s004.docx]

**S4 Table. Laboratory parameters prior to liver biopsy.**

| **Laboratory findings before procedure** | **Median [Q1, Q3]** |
| --- | --- |
| White blood cells (×10^3^/µL) | 6.7 [4.9, 8.7] |
| Platelet count (×10^3^/µL) | 226.0 [138.5, 294.0] |
| Hemoglobin (g/dL) | 12.4 [10.8, 14.0] |
| Aspartate aminotransferase (U/L) | 40.3 [23.6, 72.8] |
| Alanine aminotransferase (U/L) | 33.5 [15.9, 67.1] |
| Alkaline phosphatase (U/L) | 140.0 [86.4, 260.0] |
| Gamma-glutamyl transferase (U/L) | 119.0 [61.5, 175.0] |
| Total bilirubin (mg/dL) | 0.7 [0.4, 2.6] |
| Prothrombin time (sec) | 14.0 [13.0, 15.6] |
| International normalized ratio (INR) | 1.1 [1.0, 1.2] |

Q1: lower quartile, Q3: upper quartile
