# Supplementary material for: Complications and mortality following percutaneous and laparoscopic liver biopsy: A multicenter study in a resource‑limited healthcare system
Source: PLoS One. 2026 Apr 17;21(4):e0347300. doi: 10.1371/journal.pone.0347300 (PMC13089758; doi:10.1371/journal.pone.0347300)
Supplement: S5 Table — (DOCX) [file pone.0347300.s005.docx]

**S5 Table. Clinical impression prior to biopsy.**

| **Clinical impression prior to biopsy** | **n (%)** |
| --- | --- |
| Malignancy | 115 (52.8) |
| Hepatitis | 35 (16.1) |
| Cirrhosis | 34 (15.6) |
| Fatty liver | 13 (6.0) |
| Cholestatic liver disease | 8 (3.7) |
| Metabolic liver disease | 8 (3.7) |
| Liver injury | 3 (1.4) |
| Fibrosis | 2 (0.9) |
