# Supplementary material for: Complications and mortality following percutaneous and laparoscopic liver biopsy: A multicenter study in a resource‑limited healthcare system
Source: PLoS One. 2026 Apr 17;21(4):e0347300. doi: 10.1371/journal.pone.0347300 (PMC13089758; doi:10.1371/journal.pone.0347300)
Supplement: S6 Table — (DOCX) [file pone.0347300.s006.docx]

**S6 Table. Laboratory parameters following liver biopsy.**

| **Laboratory findings after procedure** | **Median [Q1, Q3]** |
| --- | --- |
| White blood cells (×10^3^/µL) | 7.9 [5.7, 11.1] |
| Platelet count (×10^3^/µL) | 224.0 [141.0, 295.0] |
| Hemoglobin (g/dL) | 11.5 [9.8, 13.5] |
| Alanine aminotransferase (U/L) | 36.5 [16.8, 70.0] |
| Alkaline phosphatase (U/L) | 129.0 [87.3, 280.5] |
| Gamma-glutamyl transferase (U/L) | 96.0 [40.8, 213.5] |
| Total bilirubin (mg/dL) | 0.8 [0.4, 2.5] |

Q1: lower quartile, Q3: upper quartile
