# Supplementary material for: Complications and mortality following percutaneous and laparoscopic liver biopsy: A multicenter study in a resource‑limited healthcare system
Source: PLoS One. 2026 Apr 17;21(4):e0347300. doi: 10.1371/journal.pone.0347300 (PMC13089758; doi:10.1371/journal.pone.0347300)
Supplement: S7 Table — (DOCX) [file pone.0347300.s007.docx]

**S7 Table. Bivariate analysis of factors associated with procedure-related infections.**

|  |  | **Infection** | |  |
| --- | --- | --- | --- | --- |
|  |  | **No** | **Yes** |  |
| **Variable** | **Subcategory** | **n (%) or median [Q1, Q3]** | **n (%) or median [Q1, Q3]** | **p** |
| Sex | Male, n (%) | 114 (52.3) | 4 (1.8) | 0.871 |
|  | Female, n (%) | 97 (44.5) | 3 (1.4) |  |
| Age (years) | median [Q1, Q3] | 52.0 [35.5, 62.5] | 51.0 [38.0, 66.0] | 0.824 |
| Smoking status | No, n (%) | 127 (58.3) | 5 (2.3) | 0.549 |
|  | Yes, n (%) | 84 (38.5) | 2 (0.9) |  |
| Others comorbid conditions | No, n (%) | 47 (21.6) | 1 (0.5) | 0.616 |
|  | Yes, n (%) | 164 (75.2) | 6 (2.8) |  |
| History of liver disease | No, n (%) | 161 (73.9) | 7 (3.2) | 0.142 |
|  | Yes, n (%) | 50 (22.9) | 0 (0.0) |  |
| Imaging before biopsy | No, n (%) | 27 (12.4) | 1 (0.5) | 0.908 |
|  | Yes, n (%) | 184 (84.4) | 6 (2.8) |  |
| Type of biopsy procedure | Laparoscopic, n (%) | 64 (29.4) | 0 (0.0) | 0.083 |
|  | Percutaneous, n (%) | 147 (67.4) | 7 (3.2) |  |
| Type of guidance | Direct vision, n (%) | 62 (28.4) | 0 (0.0) | 0.132 |
|  | Computed tomography, n (%) | 59 (27.1) | 4 (1.8) |  |
|  | Ultrasound, n (%) | 90 (41.3) | 3 (1.4) |  |
| Type of anesthesia | General, n (%) | 63 (28.9) | 0 (0.0) | 0.086 |
|  | Local, n (%) | 148 (67.9) | 7 (3.2) |  |
| Expected malignancy before biopsy | No, n (%) | 101 (46.3) | 2 (0.9) | 0.314 |
|  | Yes, n (%) | 110 (50.5) | 5 (2.3) |  |
| **Pre-procedure laboratory findings** |  |  |  |  |
| White blood cells (×10^3^/µL) | median [Q1, Q3] | 6.5 [4.9, 8.5] | 9.5 [8.1, 12.0] | **0.036** |
| Platelet count (×10^3^/µL) | median [Q1, Q3] | 222.0 [138.0, 289.0] | 247.0 [236.5, 431.0] | 0.094 |
| Hemoglobin (g/dL) | median [Q1, Q3] | 12.4 [10.8, 14.0] | 12.4 [9.9, 14.3] | 0.884 |
| Aspartate aminotransferase (U/L) | median [Q1, Q3] | 38.4 [23.4, 60.4] | 195.0 [101.0, 209.0] | **0.008** |
| Alanine aminotransferase (U/L) | median [Q1, Q3] | 32.7 [15.7, 60.0] | 165.0 [58.0, 345.0] | **0.021** |
| Alkaline phosphatase (U/L) | median [Q1, Q3] | 137.5 [86.0, 224.0] | 232.0 [126.0, 320.0] | 0.184 |
| Gamma-glutamyl transferase (U/L) | median [Q1, Q3] | 0.0 [0.0, 0.0] | 0.0 [0.0, 0.0] | 0.464 |
| Total bilirubin (mg/dL) | median [Q1, Q3] | 0.7 [0.4, 2.3] | 4.9 [1.5, 16.0] | 0.152 |
| Prothrombin time (sec) | median [Q1, Q3] | 14.0 [13.0, 15.6] | 13.9 [12.2, 19.4] | 0.925 |
| International normalized ratio (INR) | median [Q1, Q3] | 1.1 [1.0, 1.2] | 1.1 [1.0, 1.5] | 0.576 |

Q1: lower quartile, Q3: upper quartile, p: p-value, statistically significant p-values are in boldface
