# Supplementary material for: Complications and mortality following percutaneous and laparoscopic liver biopsy: A multicenter study in a resource‑limited healthcare system
Source: PLoS One. 2026 Apr 17;21(4):e0347300. doi: 10.1371/journal.pone.0347300 (PMC13089758; doi:10.1371/journal.pone.0347300)
Supplement: S8 Table — (DOCX) [file pone.0347300.s008.docx]

**S8 Table. Bivariate analysis of factors associated with procedure-related hemorrhage.**

|  |  | **Hemorrhage** | |  |
| --- | --- | --- | --- | --- |
|  |  | **No** | **Yes** |  |
| **Variable** | **Subcategory** | **n (%) or median [Q1, Q3]** | **n (%) or median [Q1, Q3]** | **p** |
| Sex | Male, n (%) | 115 (52.8) | 3 (1.4) | 0.837 |
|  | Female, n (%) | 97 (44.5) | 3 (1.4) |  |
| Age (years) | median [Q1, Q3] | 52.0 [35.5, 62.5] | 63.5 [29.0, 72.0] | 0.315 |
| Smoking status | No, n (%) | 127 (58.3) | 5 (2.3) | 0.247 |
|  | Yes, n (%) | 85 (39.0) | 1 (0.5) |  |
| Others comorbid conditions | No, n (%) | 47 (21.6) | 1 (0.5) | 0.748 |
|  | Yes, n (%) | 165 (75.7) | 5 (2.3) |  |
| History of liver disease | No, n (%) | 163 (74.8) | 5 (2.3) | 0.711 |
|  | Yes, n (%) | 49 (22.5) | 1 (0.5) |  |
| Imaging before biopsy | No, n (%) | 28 (12.8) | 0 (0.0) | 0.340 |
|  | Yes, n (%) | 184 (84.4) | 6 (2.8) |  |
| Type of biopsy procedure | Laparoscopic, n (%) | 60 (27.5) | 4 (1.8) | **0.042** |
|  | Percutaneous, n (%) | 152 (69.7) | 2 (0.9) |  |
| Type of guidance | Direct vision, n (%) | 58 (26.6) | 4 (1.8) | 0.107 |
|  | Computed tomography, n (%) | 62 (28.4) | 1 (0.5) |  |
|  | Ultrasound, n (%) | 92 (42.2) | 1 (0.5) |  |
| Type of anesthesia | General, n (%) | 59 (27.1) | 4 (1.8) | **0.038** |
|  | Local, n (%) | 153 (70.2) | 2 (0.9) |  |
| Expected malignancy before biopsy | No, n (%) | 101 (46.3) | 2 (0.9) | 0.489 |
|  | Yes, n (%) | 111 (50.9) | 4 (1.8) |  |
| **Pre-procedure laboratory findings** |  |  |  |  |
| White blood cells (×10^3^/µL) | median [Q1, Q3] | 6.7 [4.9, 8.7] | 6.8 [5.7, 8.4] | 0.764 |
| Platelet count (×10^3^/µL) | median [Q1, Q3] | 222.0 [138.5, 294.0] | 237.0 [169.0, 269.0] | 0.804 |
| Hemoglobin (g/dL) | median [Q1, Q3] | 12.4 [10.9, 14.0] | 10.7 [10.1, 15.1] | 0.433 |
| Aspartate aminotransferase | median [Q1, Q3] | 40.0 [24.0, 72.0] | 40.8 [29.0, 124.9] | 0.954 |
| Alanine aminotransferase (U/L) | median [Q1, Q3] | 33.3 [16.0, 60.0] | 64.2 [39.6, 204.6] | 0.489 |
| Alkaline phosphatase (U/L) | median [Q1, Q3] | 137.5 [88.2, 246.0] | 160.0 [116.0, 513.0] | 0.608 |
| Total bilirubin (mg/dL) | median [Q1, Q3] | 0.7 [0.4, 2.6] | 0.6 [0.5, 3.9] | 0.939 |
| Prothrombin time (sec) | median [Q1, Q3] | 14.0 [13.0, 15.6] | 14.2 [13.5, 17.1] | 0.659 |
| International normalized ratio (INR) | median [Q1, Q3] | 1.1 [1.0, 1.2] | 1.1 [1.0, 1.3] | 0.769 |

Q1: lower quartile, Q3: upper quartile, p: p-value, statistically significant p-values are in boldface
