# Supplementary material for: Complications and mortality following percutaneous and laparoscopic liver biopsy: A multicenter study in a resource‑limited healthcare system
Source: PLoS One. 2026 Apr 17;21(4):e0347300. doi: 10.1371/journal.pone.0347300 (PMC13089758; doi:10.1371/journal.pone.0347300)
Supplement: S9 Table — (DOCX) [file pone.0347300.s009.docx]

**S9 Table. Bivariate analysis of factors associated with procedure-related pulmonary complications.**

|  |  | **Pulmonary complications** | |  |
| --- | --- | --- | --- | --- |
|  |  | **No** | **Yes** |  |
| **Variable** | **Subcategory** | **n (%) or median [Q1, Q3]** | **n (%) or median [Q1, Q3]** | **p** |
| Sex | Male, n (%) | 116 (53.2) | 2 (0.9) | 0.867 |
|  | Female, n (%) | 98 (45.0) | 2 (0.9) |  |
| Age (years) | median [Q1, Q3] | 52.0 [35.0, 63.0] | 62.5 [46.0, 72.5] | 0.228 |
| Smoking status | No, n (%) | 128 (58.7) | 4 (1.8) | 0.103 |
|  | Yes, n (%) | 86 (39.4) | 0 (0.0) |  |
| Others comorbid conditions | No, n (%) | 48 (22.0) | 0 (0.0) | 0.283 |
|  | Yes, n (%) | 166 (76.1) | 4 (1.8) |  |
| History of liver disease | No, n (%) | 165 (75.7) | 3 (1.4) | 0.921 |
|  | Yes, n (%) | 49 (22.5) | 1 (0.5) |  |
| Imaging before biopsy | No, n (%) | 28 (12.8) | 0 (0.0) | 0.438 |
|  | Yes, n (%) | 186 (85.3) | 4 (1.8) |  |
| Type of biopsy procedure | Laparoscopic, n (%) | 61 (28.0) | 3 (1.4) | **0.043** |
|  | Percutaneous, n (%) | 153 (70.2) | 1 (0.5) |  |
| Type of guidance | Direct vision, n (%) | 59 (27.1) | 3 (1.4) | 0.101 |
|  | Computed tomography, n (%) | 63 (28.9) | 0 (0.0) |  |
|  | Ultrasound, n (%) | 92 (42.2) | 1 (0.5) |  |
| Type of anesthesia | General, n (%) | 60 (27.5) | 3 (1.4) | **0.040** |
|  | Local, n (%) | 154 (70.6) | 1 (0.5) |  |
| Expected malignancy before biopsy | No, n (%) | 102 (46.8) | 1 (0.5) | 0.368 |
|  | Yes, n (%) | 112 (51.4) | 3 (1.4) |  |
| **Pre-procedure laboratory findings** |  |  |  |  |
| White blood cells (×10^3^/µL) | median [Q1, Q3] | 6.7 [4.9, 8.6] | 7.0 [5.1, 10.3] | 0.867 |
| Platelet count (×10^3^/µL) | median [Q1, Q3] | 222.0 [138.0, 292.0] | 254.4 [207.4, 398.5] | 0.326 |
| Hemoglobin (g/dL) | median [Q1, Q3] | 12.4 [10.9, 14.0] | 9.5 [8.5, 11.7] | 0.063 |
| Aspartate aminotransferase | median [Q1, Q3] | 40.7 [25.2, 72.5] | 13.7 [11.6, 53.9] | 0.065 |
| Alanine aminotransferase (U/L) | median [Q1, Q3] | 34.3 [16.0, 66.1] | 12.2 [6.2, 44.1] | 0.151 |
| Alkaline phosphatase (U/L) | median [Q1, Q3] | 137.5 [88.2, 228.0] | 410.0 [231.5, 452.0] | 0.521 |
| Gamma-glutamyl transferase (U/L) | median [Q1, Q3] | 114.0 [70.0, 165.0] | 222.0 [130.0, 307.5] | 0.467 |
| Total bilirubin (mg/dL) | median [Q1, Q3] | 0.7 [0.4, 2.8] | 0.5 [0.4, 0.7] | 0.336 |
| Prothrombin time (sec) | median [Q1, Q3] | 14.0 [13.0, 15.6] | 14.2 [13.5, 14.5] | 0.754 |
| International normalized ratio (INR) | median [Q1, Q3] | 1.1 [1.0, 1.2] | 1.1 [1.0, 1.1] | 0.632 |

Q1: lower quartile, Q3: upper quartile, p: p-value, statistically significant p-values are in boldface
