# Supplementary material for: Complications and mortality following percutaneous and laparoscopic liver biopsy: A multicenter study in a resource‑limited healthcare system
Source: PLoS One. 2026 Apr 17;21(4):e0347300. doi: 10.1371/journal.pone.0347300 (PMC13089758; doi:10.1371/journal.pone.0347300)
Supplement: S10 Table — (DOCX) [file pone.0347300.s010.docx]

**S10 Table. Bivariate analysis of factors associated with mortality.**

|  |  | **Mortality** | |  |
| --- | --- | --- | --- | --- |
|  |  | **No** | **Yes** |  |
| **Variable** | **Subcategory** | **n (%) or median [Q1, Q3]** | **n (%) or median [Q1, Q3]** | **p** |
| Sex | Male, n (%) | 115 (52.8) | 3 (1.4) | 0.837 |
|  | Female, n (%) | 97 (44.5) | 3 (1.4) |  |
| Age (years) | median [Q1, Q3] | 52.0 [35.0, 62.0] | 65.5 [56.0, 69.0] | **0.035** |
| Smoking status | No, n (%) | 127 (58.3) | 5 (2.3) | 0.247 |
|  | Yes, n (%) | 85 (39.0) | 1 (0.5) |  |
| Others comorbid conditions | No, n (%) | 48 (22.0) | 0 (0.0) | 0.187 |
|  | Yes, n (%) | 164 (75.2) | 6 (2.8) |  |
| History of liver disease | No, n (%) | 162 (74.3) | 6 (2.8) | 0.175 |
|  | Yes, n (%) | 50 (22.9) | 0 (0.0) |  |
| Imaging before biopsy | No, n (%) | 27 (12.4) | 1 (0.5) | 0.777 |
|  | Yes, n (%) | 185 (84.9) | 5 (2.3) |  |
| Type of biopsy procedure | Laparoscopic, n (%) | 63 (28.9) | 1 (0.5) | 0.489 |
|  | Percutaneous, n (%) | 149 (68.3) | 5 (2.3) |  |
| Type of guidance | Direct vision, n (%) | 61 (28.0) | 1 (0.5) | 0.502 |
|  | Computed tomography, n (%) | 60 (27.5) | 3 (1.4) |  |
|  | Ultrasound, n (%) | 91 (41.7) | 2 (0.9) |  |
| Type of anesthesia | General, n (%) | 62 (28.4) | 1 (0.5) | 0.503 |
|  | Local, n (%) | 150 (68.8) | 5 (2.3) |  |
| Expected malignancy before biopsy | No, n (%) | 102 (46.8) | 1 (0.5) | 0.128 |
|  | Yes, n (%) | 110 (50.5) | 5 (2.3) |  |
| Infection | No, n (%) | 208 (95.4) | 3 (1.4) | **< 0.001** |
|  | Yes, n (%) | 4 (1.8) | 3 (1.4) |  |
| Hemorrhage | No, n (%) | 207 (95.0) | 5 (2.3) | **0.035** |
|  | Yes, n (%) | 5 (2.3) | 1 (0.5) |  |
| Pulmonary | No, n (%) | 210 (96.3) | 4 (1.8) | **< 0.001** |
|  | Yes, n (%) | 2 (0.9) | 2 (0.9) |  |
| Metabolic disturbances | No, n (%) | 211 (96.8) | 4 (1.8) | **< 0.001** |
|  | Yes, n (%) | 1 (0.5) | 2 (0.9) |  |
| Acute kidney injury | No, n (%) | 212 (97.2) | 4 (1.8) | **< 0.001** |
|  | Yes, n (%) | 0 (0.0) | 2 (0.9) |  |
| Other complications | No, n (%) | 211 (96.8) | 5 (2.3) | **< 0.001** |
|  | Yes, n (%) | 1 (0.5) | 1 (0.5) |  |
| Post-biopsy imaging performed | No, n (%) | 203 (93.1) | 3 (1.4) | **< 0.001** |
|  | Yes, n (%) | 9 (4.1) | 3 (1.4) |  |
| **Pre-procedure laboratory findings** |  |  |  |  |
| White blood cells (×10^3^/µL) | median [Q1, Q3] | 6.5 [4.8, 8.6] | 9.3 [7.5, 13.1] | 0.051 |
| Platelet count (×10^3^/µL) | median [Q1, Q3] | 222.0 [138.0, 290.5] | 239.0 [236.8, 461.0] | 0.082 |
| Hemoglobin (g/dL) | median [Q1, Q3] | 12.4 [10.9, 14.0] | 9.9 [7.9, 11.1] | 0.097 |
| Aspartate aminotransferase | median [Q1, Q3] | 39.9 [23.8, 60.9] | 92.7 [60.0, 101.0] | 0.188 |
| Alanine aminotransferase (U/L) | median [Q1, Q3] | 33.7 [16.0, 62.1] | 33.0 [16.7, 71.5] | 0.816 |
| Alkaline phosphatase (U/L) | median [Q1, Q3] | 133.0 [86.2, 210.0] | 452.0 [321.0, 680.0] | **0.008** |
| Gamma-glutamyl transferase (U/L) | median [Q1, Q3] | 109.0 [61.5, 163.5] | 307.5 [222.0, 393.0] | 0.078 |
| Total bilirubin (mg/dL) | median [Q1, Q3] | 0.7 [0.4, 2.6] | 0.8 [0.7, 4.0] | 0.442 |
| Prothrombin time (sec) | median [Q1, Q3] | 14.0 [13.0, 15.6] | 13.2 [12.1, 14.5] | 0.258 |
| International normalized ratio (INR) | median [Q1, Q3] | 1.1 [1.0, 1.2] | 1.1 [0.9, 1.1] | 0.601 |
| **Post-procedure laboratory findings** |  |  |  |  |
| White blood cells (×10^3^/µL) | median [Q1, Q3] | 7.7 [5.6, 10.8] | 12.6 [11.5, 15.0] | **0.004** |
| Platelet count (×10^3^/µL) | median [Q1, Q3] | 218.6 [136.5, 292.0] | 260.5 [245.1, 351.0] | 0.101 |
| Hemoglobin (g/dL) | median [Q1, Q3] | 11.6 [9.9, 13.5] | 10.2 [8.3, 11.2] | 0.111 |
| Alanine aminotransferase (U/L) | median [Q1, Q3] | 34.7 [16.0, 59.5] | 79.5 [74.2, 104.0] | **0.033** |
| Alkaline phosphatase (U/L) | median [Q1, Q3] | 121.0 [86.3, 237.0] | 452.0 [427.0, 552.0] | **0.004** |
| Gamma-glutamyl transferase (U/L) | median [Q1, Q3] | 86.3 [40.6, 144.0] | 445.0 [410.5, 513.5] | **0.019** |
| Total bilirubin (mg/dL) | median [Q1, Q3] | 0.7 [0.4, 2.2] | 2.4 [1.7, 7.2] | 0.080 |

Q1: lower quartile, Q3: upper quartile, p: p-value, statistically significant p-values are in boldface
